# Methyl (*S*_p_)-2-(diphenyl­phosphino)ferrocene-1-carboxyl­ate

**DOI:** 10.1107/S1600536809036654

**Published:** 2009-09-16

**Authors:** Petr Štěpnička, Jiří Tauchman, Ivana Císařová

**Affiliations:** a Department of Inorganic Chemistry, Faculty of Science, Charles University in Prague; Hlavova 2030, 12840 Prague 2, Czech Republic

## Abstract

The title compound, [Fe(C_5_H_5_)(C_19_H_16_O_2_P)], obtained serendipitously during recrystallization of 1-hydroxy­benzotriazolyl (*S*_p_)-2-(diphenyl­phosphino)ferrocene-1-carboxyl­ate from meth­anol, crystallizes in the chiral space group *P*2_1_2_1_2_1_. Its crystal structure not only confirms the anti­cipated absolute configuration but also establishes a rather regular geometry for the ferrocene unit, devoid of any significant deformation due to the attached substituents. In the crystal, symmetry-related mol­ecules are linked *via* weak C—H⋯O inter­actions.

## Related literature

For an overview of the chemistry of ferrocene, see: Štěpnička (2008 ▶). For the NMR spectroscopic data of the title compound, see: You *et al.* (2002 ▶); Lamač *et al.* (2008 ▶). For the structure of similar compounds, see: Lamač *et al.* (2009 ▶); Štěpnička (2002 ▶).
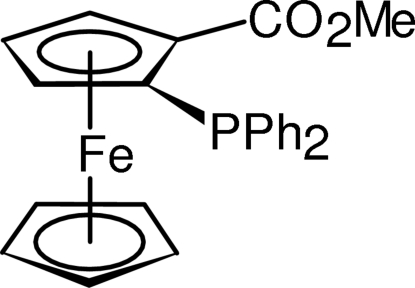


## Experimental

### 

#### Crystal data

[Fe(C_5_H_5_)(C_19_H_16_O_2_P)]
*M* *_r_* = 428.23
Orthorhombic, 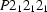

*a* = 10.6867 (2) Å
*b* = 12.9015 (3) Å
*c* = 14.1042 (2) Å
*V* = 1944.61 (6) Å^3^
*Z* = 4
Mo *K*α radiation
μ = 0.88 mm^−1^
*T* = 150 K
0.40 × 0.40 × 0.28 mm

#### Data collection

Nonius KappaCCD diffractometer
Absorption correction: none
15400 measured reflections
4449 independent reflections
4228 reflections with *I* > 2σ(*I*)
*R*_int_ = 0.036

#### Refinement

*R*[*F* ^2^ > 2σ(*F* ^2^)] = 0.025
*wR*(*F* ^2^) = 0.060
*S* = 1.06
4449 reflections
254 parameters
H-atom parameters constrained
Δρ_max_ = 0.51 e Å^−3^
Δρ_min_ = −0.29 e Å^−3^
Absolute structure: Flack (1983 ▶), 1918 Friedel pairs
Flack parameter: 0.004 (11)

### 

Data collection: *COLLECT* (Nonius, 2000 ▶); cell refinement: *HKL* *SCALEPACK* (Otwinowski & Minor, 1997 ▶); data reduction: *HKL* *DENZO* (Otwinowski & Minor, 1997 ▶) and *SCALEPACK*; program(s) used to solve structure: *SIR97* (Altomare *et al*., 1999 ▶); program(s) used to refine structure: *SHELXL97* (Sheldrick, 2008 ▶); molecular graphics: *PLATON* (Spek, 2009 ▶); software used to prepare material for publication: *SHELXL97* and *PLATON*.

## Supplementary Material

Crystal structure: contains datablocks I, global. DOI: 10.1107/S1600536809036654/su2145sup1.cif

Structure factors: contains datablocks I. DOI: 10.1107/S1600536809036654/su2145Isup2.hkl

Additional supplementary materials:  crystallographic information; 3D view; checkCIF report

## Figures and Tables

**Table 1:** Hydrogen-bond geometry (Å, °)

*D*—H⋯*A*	*D*—H	H⋯*A*	*D*⋯*A*	*D*—H⋯*A*
C4—H4⋯O1^i^	0.93	2.58	3.184 (2)	123
C15—H15⋯O1^ii^	0.93	2.58	3.494 (3)	167

Symmetry codes: (i) 
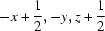
; (ii) 
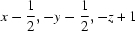
.

**Fig. 1.**
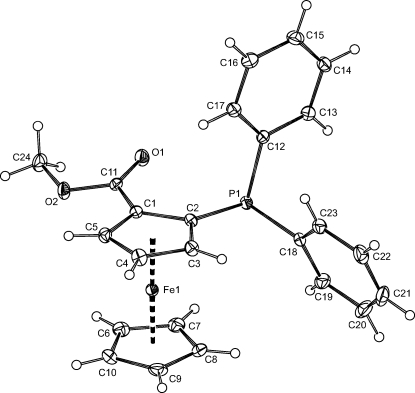
View of the molecular structure of the title compound, showing the atom numbering scheme and displacement ellipsoids drawn at the 30% probability level.

## supplementary crystallographic information

### Comment

The crystal structure of the title compound was determined at 150 (2) K (Fig. 1). It crystallizes with the symmetry of the chiral space group *P*2_1_2_1_2_1_ and one molecule in the asymmetric unit. The molecular geometry of the title compound compares well with the data reported previously for (*S*_p_)-2-(diphenylphosphinoyl)ferrocene-1-carboxylic acid (Štěpnička, 2002) and methyl (*R*_p_)-1',2-bis(diphenylphosphino)ferrocene-1-carboxylate (Lamač *et al.*, 2009). The geometry of the ferrocene moiety is quite regular, showing similar Fe—ring centroid distances (1.6471 (9) and 1.6567 (9) Å for the rings C(1–5) and C(6–10), respectively) and insignificant tilting (the dihedral angle of the cyclopentadienyl mean planes being 2.08 (12) °). The attached substituents do not seem to impose any pronounced deformation of the ferrocene core, as evidenced by the C11—C1—C2—P torsion angle of –5.5 (3) °. However, the diphenylphosphinyl group binds somewhat unsymmetrically as indicated by the differences in the C(1/3)—C2—P angles being *ca* 2.7 ° (*cf.* the difference of the C(2/5)—C1—C11 angles, which is below 0.1 °, and also the perpendicular distances from the C(1–5) ring mean-plane: 0.136 (1) Å for P and –0.018 (2) Å for C11). This is reflected also by the variation in the Fe–C distances (2.0283 (18)–2.0672 (18) Å) for the substituted cyclopentadienyl ring (C1–C5).

The crystal structure of the title compound is essentially molecular, with symmetry related molecules forming only soft C—H···O contacts (Table 1).

### Experimental

The title compound was formed on recrystallization of 1-hydroxybenzotriazolyl (*S*_p_)-2-(diphenylphosphino)ferrocene-1-carboxylate (Štěpnička & Tauchman, unpublished results) from warm methanol, apparently resulting from a trans-esterification reaction of the starting activated ester. Its formulation was established by NMR spectroscopy (You *et al.*, 2002; Lamač *et al.*, 2008).

### Refinement

The H-atoms were included in calculated positions and treated as riding atoms: C-H = 0.93 (aromatic CH) and 0.96 (CH_3_) Å, and *U*_iso_(H) = k × *U*_eq_(parent C-atom), where k = 1.2 for aromatic CH, and 1.5 for CH_3_.

### Crystal data

**Table:**

[Fe(C_5_H_5_)(C_19_H_16_O_2_P)]	*F*(000) = 888
*M**_r_* = 428.23	*D*_x_ = 1.463 Mg m^−^^3^
Orthorhombic, *P*2_1_2_1_2_1_	Mo *K*α radiation, λ = 0.71073 Å
Hall symbol: P 2ac 2ab	Cell parameters from 2517 reflections
*a* = 10.6867 (2) Å	θ = 1.0–27.5°
*b* = 12.9015 (3) Å	µ = 0.88 mm^−^^1^
*c* = 14.1042 (2) Å	*T* = 150 K
*V* = 1944.61 (6) Å^3^	Block, orange
*Z* = 4	0.40 × 0.40 × 0.28 mm

### Data collection

**Table:**

Nonius KappaCCD diffractometer	4228 reflections with *I* > 2σ(*I*)
Radiation source: fine-focus sealed tube	*R*_int_ = 0.036
horizontally mounted graphite crystal	θ_max_ = 27.5°, θ_min_ = 2.1°
Detector resolution: 9.091 pixels mm^-1^	*h* = −13→13
ω and π scans to fill the Ewald sphere	*k* = −16→16
15400 measured reflections	*l* = −18→18
4449 independent reflections	

### Refinement

**Table:**

Refinement on *F*^2^	Secondary atom site location: difference Fourier map
Least-squares matrix: full	Hydrogen site location: inferred from neighbouring sites
*R*[*F*^2^ > 2σ(*F*^2^)] = 0.025	H-atom parameters constrained
*wR*(*F*^2^) = 0.060	*w* = 1/[σ^2^(*F*_o_^2^) + (0.0238*P*)^2^ + 0.8124*P*] where *P* = (*F*_o_^2^ + 2*F*_c_^2^)/3
*S* = 1.06	(Δ/σ)_max_ = 0.001
4449 reflections	Δρ_max_ = 0.51 e Å^−^^3^
254 parameters	Δρ_min_ = −0.29 e Å^−^^3^
0 restraints	Absolute structure: Flack (1983), 1918 Friedel pairs
Primary atom site location: structure-invariant direct methods	Flack parameter: 0.004 (11)

### Special details

**Table:**

Geometry. Bond distances, angles etc. have been calculated using the rounded fractional coordinates. All su's are estimated from the variances of the (full) variance-covariance matrix. The cell esds are taken into account in the estimation of distances, angles and torsion angles
Refinement. Refinement of *F*^2^ against ALL reflections. The weighted *R*-factor *wR* and goodness of fit *S* are based on *F*^2^, conventional *R*-factors *R* are based on *F*, with *F* set to zero for negative *F*^2^. The threshold expression of *F*^2^ > σ(*F*^2^) is used only for calculating *R*-factors(gt) *etc*. and is not relevant to the choice of reflections for refinement. *R*-factors based on *F*^2^ are statistically about twice as large as those based on *F*, and *R*- factors based on ALL data will be even larger.

### Fractional atomic coordinates and isotropic or equivalent isotropic displacement parameters (Å^2^)

**Table:**

	*x*	*y*	*z*	*U*_iso_*/*U*_eq_	
Fe1	0.29965 (2)	0.18843 (2)	0.72459 (2)	0.0181 (1)	
P1	0.09939 (4)	0.08083 (4)	0.56683 (3)	0.0184 (1)	
O1	0.36618 (12)	0.01291 (12)	0.50125 (10)	0.0306 (4)	
O2	0.53342 (11)	0.03569 (11)	0.59516 (9)	0.0268 (4)	
C1	0.33684 (16)	0.04902 (14)	0.66438 (12)	0.0188 (5)	
C2	0.20328 (18)	0.06630 (13)	0.66896 (11)	0.0184 (4)	
C3	0.17351 (16)	0.07818 (15)	0.76755 (13)	0.0224 (5)	
C4	0.28491 (19)	0.06905 (15)	0.82171 (12)	0.0252 (5)	
C5	0.38563 (17)	0.05158 (14)	0.75918 (13)	0.0224 (5)	
C6	0.40940 (19)	0.30253 (16)	0.66633 (14)	0.0273 (6)	
C7	0.28422 (19)	0.31090 (16)	0.63399 (13)	0.0284 (5)	
C8	0.20585 (19)	0.32594 (14)	0.71400 (16)	0.0318 (6)	
C9	0.2832 (2)	0.32638 (16)	0.79572 (14)	0.0322 (6)	
C10	0.40893 (18)	0.31174 (16)	0.76638 (14)	0.0284 (5)	
C11	0.40972 (16)	0.03148 (13)	0.57782 (13)	0.0192 (5)	
C12	0.04477 (16)	−0.05296 (14)	0.54954 (12)	0.0188 (5)	
C13	−0.04839 (17)	−0.06903 (16)	0.48155 (12)	0.0221 (5)	
C14	−0.09729 (19)	−0.16720 (16)	0.46657 (13)	0.0268 (5)	
C15	−0.0535 (2)	−0.25121 (17)	0.51784 (15)	0.0304 (6)	
C16	0.0408 (2)	−0.23636 (16)	0.58396 (15)	0.0308 (6)	
C17	0.08946 (18)	−0.13788 (15)	0.59981 (14)	0.0249 (5)	
C18	−0.04185 (16)	0.13315 (15)	0.62459 (13)	0.0215 (5)	
C19	−0.0773 (2)	0.23452 (16)	0.60378 (15)	0.0305 (6)	
C20	−0.1852 (2)	0.2765 (2)	0.64381 (16)	0.0438 (8)	
C21	−0.2570 (2)	0.2184 (2)	0.70388 (16)	0.0431 (8)	
C22	−0.22483 (18)	0.11714 (19)	0.72475 (16)	0.0371 (7)	
C23	−0.11703 (17)	0.07494 (17)	0.68559 (14)	0.0278 (5)	
C24	0.61344 (18)	0.01350 (18)	0.51607 (14)	0.0309 (6)	
H3	0.09400	0.09000	0.79200	0.0270*	
H4	0.29050	0.07380	0.88740	0.0300*	
H5	0.46900	0.04320	0.77630	0.0270*	
H6	0.47960	0.29270	0.62840	0.0330*	
H7	0.25800	0.30720	0.57120	0.0340*	
H8	0.11940	0.33400	0.71300	0.0380*	
H9	0.25610	0.33480	0.85790	0.0390*	
H10	0.47860	0.30870	0.80580	0.0340*	
H13	−0.07780	−0.01340	0.44600	0.0270*	
H14	−0.16000	−0.17670	0.42170	0.0320*	
H15	−0.08690	−0.31690	0.50800	0.0360*	
H16	0.07170	−0.29260	0.61790	0.0370*	
H17	0.15250	−0.12880	0.64450	0.0300*	
H19	−0.02870	0.27440	0.56300	0.0370*	
H20	−0.20840	0.34430	0.62960	0.0530*	
H21	−0.32830	0.24730	0.73100	0.0520*	
H22	−0.27500	0.07760	0.76470	0.0450*	
H23	−0.09470	0.00710	0.70020	0.0330*	
H24A	0.61180	−0.05950	0.50310	0.0460*	
H24B	0.69750	0.03430	0.53080	0.0460*	
H24C	0.58460	0.05090	0.46140	0.0460*	

### Atomic displacement parameters (Å^2^)

**Table:**

	*U*^11^	*U*^22^	*U*^33^	*U*^12^	*U*^13^	*U*^23^
Fe1	0.0169 (1)	0.0204 (1)	0.0170 (1)	−0.0002 (1)	0.0003 (1)	−0.0020 (1)
P1	0.0167 (2)	0.0190 (2)	0.0196 (2)	−0.0010 (2)	−0.0012 (2)	0.0012 (2)
O1	0.0238 (7)	0.0435 (9)	0.0244 (6)	0.0042 (6)	−0.0028 (6)	−0.0110 (6)
O2	0.0157 (6)	0.0392 (8)	0.0256 (7)	0.0010 (6)	0.0011 (5)	−0.0030 (6)
C1	0.0189 (8)	0.0166 (9)	0.0210 (8)	0.0004 (7)	−0.0013 (7)	0.0004 (7)
C2	0.0173 (8)	0.0189 (8)	0.0190 (7)	−0.0036 (8)	−0.0004 (7)	0.0007 (6)
C3	0.0190 (8)	0.0273 (9)	0.0208 (8)	−0.0038 (7)	0.0048 (7)	0.0011 (8)
C4	0.0271 (9)	0.0311 (10)	0.0175 (8)	−0.0027 (8)	0.0000 (8)	0.0052 (7)
C5	0.0217 (8)	0.0231 (9)	0.0225 (9)	0.0013 (7)	−0.0042 (7)	0.0035 (7)
C6	0.0283 (10)	0.0227 (10)	0.0309 (9)	−0.0052 (9)	0.0065 (8)	0.0011 (8)
C7	0.0367 (10)	0.0191 (8)	0.0293 (9)	0.0027 (9)	−0.0063 (8)	0.0033 (8)
C8	0.0259 (9)	0.0198 (9)	0.0497 (12)	0.0048 (8)	−0.0013 (10)	−0.0072 (9)
C9	0.0371 (11)	0.0279 (10)	0.0316 (10)	−0.0041 (9)	0.0078 (9)	−0.0133 (8)
C10	0.0270 (9)	0.0267 (9)	0.0315 (9)	−0.0060 (9)	−0.0048 (8)	−0.0047 (9)
C11	0.0181 (8)	0.0152 (8)	0.0244 (8)	0.0025 (7)	−0.0003 (7)	0.0002 (7)
C12	0.0161 (8)	0.0224 (9)	0.0178 (8)	0.0000 (7)	0.0034 (6)	−0.0025 (7)
C13	0.0232 (8)	0.0250 (10)	0.0181 (8)	0.0009 (8)	0.0009 (7)	−0.0007 (7)
C14	0.0222 (8)	0.0321 (11)	0.0261 (9)	−0.0021 (8)	−0.0012 (8)	−0.0074 (8)
C15	0.0297 (10)	0.0238 (10)	0.0376 (12)	−0.0064 (8)	0.0043 (9)	−0.0061 (9)
C16	0.0337 (11)	0.0218 (10)	0.0370 (11)	0.0019 (8)	−0.0023 (9)	0.0028 (9)
C17	0.0253 (9)	0.0233 (9)	0.0261 (9)	−0.0001 (8)	−0.0031 (8)	−0.0003 (7)
C18	0.0160 (8)	0.0236 (9)	0.0250 (9)	0.0000 (7)	−0.0041 (7)	−0.0048 (7)
C19	0.0332 (11)	0.0276 (10)	0.0307 (10)	0.0077 (9)	−0.0088 (9)	−0.0023 (8)
C20	0.0474 (14)	0.0429 (13)	0.0412 (12)	0.0272 (12)	−0.0170 (11)	−0.0124 (10)
C21	0.0252 (10)	0.0666 (17)	0.0374 (12)	0.0200 (11)	−0.0078 (9)	−0.0214 (11)
C22	0.0213 (9)	0.0546 (14)	0.0354 (11)	−0.0048 (9)	0.0035 (9)	−0.0135 (11)
C23	0.0193 (8)	0.0300 (10)	0.0342 (10)	−0.0030 (8)	0.0033 (8)	−0.0073 (8)
C24	0.0201 (9)	0.0393 (12)	0.0332 (10)	0.0027 (8)	0.0069 (8)	−0.0014 (9)

### Geometric parameters (Å, °)

**Table:**

Fe1—C1	2.0283 (18)	C14—C15	1.384 (3)
Fe1—C2	2.0394 (17)	C15—C16	1.386 (3)
Fe1—C3	2.0512 (18)	C16—C17	1.391 (3)
Fe1—C4	2.0672 (18)	C18—C19	1.393 (3)
Fe1—C5	2.0493 (18)	C18—C23	1.396 (3)
Fe1—C6	2.054 (2)	C19—C20	1.394 (3)
Fe1—C7	2.039 (2)	C20—C21	1.367 (3)
Fe1—C8	2.0431 (19)	C21—C22	1.383 (4)
Fe1—C9	2.051 (2)	C22—C23	1.389 (3)
Fe1—C10	2.060 (2)	C3—H3	0.9300
P1—C2	1.8283 (18)	C4—H4	0.9300
P1—C12	1.8384 (19)	C5—H5	0.9300
P1—C18	1.8433 (18)	C6—H6	0.9300
O1—C11	1.200 (2)	C7—H7	0.9300
O2—C11	1.346 (2)	C8—H8	0.9300
O2—C24	1.434 (2)	C9—H9	0.9300
C1—C2	1.446 (3)	C10—H10	0.9300
C1—C5	1.436 (2)	C13—H13	0.9300
C1—C11	1.466 (2)	C14—H14	0.9300
C2—C3	1.435 (2)	C15—H15	0.9300
C3—C4	1.419 (3)	C16—H16	0.9300
C4—C5	1.410 (3)	C17—H17	0.9300
C6—C7	1.418 (3)	C19—H19	0.9300
C6—C10	1.416 (3)	C20—H20	0.9300
C7—C8	1.419 (3)	C21—H21	0.9300
C8—C9	1.418 (3)	C22—H22	0.9300
C9—C10	1.419 (3)	C23—H23	0.9300
C12—C13	1.398 (2)	C24—H24A	0.9600
C12—C17	1.390 (3)	C24—H24B	0.9600
C13—C14	1.386 (3)	C24—H24C	0.9600
			
C1—Fe1—C2	41.65 (7)	Fe1—C8—C9	70.01 (12)
C1—Fe1—C3	68.75 (7)	C7—C8—C9	107.63 (18)
C1—Fe1—C4	68.39 (7)	Fe1—C9—C8	69.45 (11)
C1—Fe1—C5	41.22 (7)	Fe1—C9—C10	70.15 (12)
C1—Fe1—C6	110.87 (8)	C8—C9—C10	108.33 (17)
C1—Fe1—C7	116.15 (7)	Fe1—C10—C6	69.64 (12)
C1—Fe1—C8	146.67 (8)	Fe1—C10—C9	69.47 (11)
C1—Fe1—C9	172.42 (8)	C6—C10—C9	107.77 (17)
C1—Fe1—C10	133.92 (7)	O1—C11—O2	123.54 (16)
C2—Fe1—C3	41.06 (7)	O1—C11—C1	125.06 (16)
C2—Fe1—C4	68.95 (7)	O2—C11—C1	111.37 (15)
C2—Fe1—C5	69.66 (7)	P1—C12—C13	117.16 (14)
C2—Fe1—C6	133.49 (7)	P1—C12—C17	124.29 (14)
C2—Fe1—C7	108.47 (7)	C13—C12—C17	118.55 (17)
C2—Fe1—C8	113.27 (8)	C12—C13—C14	120.57 (18)
C2—Fe1—C9	144.64 (8)	C13—C14—C15	120.55 (18)
C2—Fe1—C10	173.33 (7)	C14—C15—C16	119.3 (2)
C3—Fe1—C4	40.32 (7)	C15—C16—C17	120.41 (19)
C3—Fe1—C5	68.09 (7)	C12—C17—C16	120.64 (18)
C3—Fe1—C6	172.11 (8)	P1—C18—C19	118.26 (14)
C3—Fe1—C7	132.02 (8)	P1—C18—C23	123.13 (15)
C3—Fe1—C8	107.54 (8)	C19—C18—C23	118.58 (17)
C3—Fe1—C9	113.64 (8)	C18—C19—C20	120.3 (2)
C3—Fe1—C10	145.46 (8)	C19—C20—C21	120.2 (2)
C4—Fe1—C5	40.05 (7)	C20—C21—C22	120.7 (2)
C4—Fe1—C6	147.42 (8)	C21—C22—C23	119.5 (2)
C4—Fe1—C7	170.60 (8)	C18—C23—C22	120.8 (2)
C4—Fe1—C8	131.15 (8)	Fe1—C3—H3	127.00
C4—Fe1—C9	108.42 (8)	C2—C3—H3	125.00
C4—Fe1—C10	115.41 (8)	C4—C3—H3	125.00
C5—Fe1—C6	117.18 (8)	Fe1—C4—H4	127.00
C5—Fe1—C7	148.56 (8)	C3—C4—H4	126.00
C5—Fe1—C8	170.22 (8)	C5—C4—H4	126.00
C5—Fe1—C9	132.05 (8)	Fe1—C5—H5	126.00
C5—Fe1—C10	110.07 (8)	C1—C5—H5	126.00
C6—Fe1—C7	40.53 (8)	C4—C5—H5	126.00
C6—Fe1—C8	68.20 (8)	Fe1—C6—H6	126.00
C6—Fe1—C9	67.83 (8)	C7—C6—H6	126.00
C6—Fe1—C10	40.28 (8)	C10—C6—H6	126.00
C7—Fe1—C8	40.67 (8)	Fe1—C7—H7	126.00
C7—Fe1—C9	68.10 (8)	C6—C7—H7	126.00
C7—Fe1—C10	68.08 (8)	C8—C7—H7	126.00
C8—Fe1—C9	40.54 (8)	Fe1—C8—H8	126.00
C8—Fe1—C10	68.19 (8)	C7—C8—H8	126.00
C9—Fe1—C10	40.38 (8)	C9—C8—H8	126.00
C2—P1—C12	101.60 (8)	Fe1—C9—H9	126.00
C2—P1—C18	100.75 (8)	C8—C9—H9	126.00
C12—P1—C18	98.19 (8)	C10—C9—H9	126.00
C11—O2—C24	115.87 (14)	Fe1—C10—H10	126.00
Fe1—C1—C2	69.59 (10)	C6—C10—H10	126.00
Fe1—C1—C5	70.17 (10)	C9—C10—H10	126.00
Fe1—C1—C11	126.14 (13)	C12—C13—H13	120.00
C2—C1—C5	108.26 (15)	C14—C13—H13	120.00
C2—C1—C11	125.84 (15)	C13—C14—H14	120.00
C5—C1—C11	125.90 (16)	C15—C14—H14	120.00
Fe1—C2—P1	122.04 (9)	C14—C15—H15	120.00
Fe1—C2—C1	68.77 (10)	C16—C15—H15	120.00
Fe1—C2—C3	69.91 (10)	C15—C16—H16	120.00
P1—C2—C1	125.45 (12)	C17—C16—H16	120.00
P1—C2—C3	128.17 (14)	C12—C17—H17	120.00
C1—C2—C3	106.18 (15)	C16—C17—H17	120.00
Fe1—C3—C2	69.03 (10)	C18—C19—H19	120.00
Fe1—C3—C4	70.45 (11)	C20—C19—H19	120.00
C2—C3—C4	109.07 (15)	C19—C20—H20	120.00
Fe1—C4—C3	69.24 (11)	C21—C20—H20	120.00
Fe1—C4—C5	69.29 (10)	C20—C21—H21	120.00
C3—C4—C5	108.48 (15)	C22—C21—H21	120.00
Fe1—C5—C1	68.61 (10)	C21—C22—H22	120.00
Fe1—C5—C4	70.66 (11)	C23—C22—H22	120.00
C1—C5—C4	108.00 (16)	C18—C23—H23	120.00
Fe1—C6—C7	69.17 (12)	C22—C23—H23	120.00
Fe1—C6—C10	70.09 (12)	O2—C24—H24A	109.00
C7—C6—C10	108.11 (17)	O2—C24—H24B	109.00
Fe1—C7—C6	70.30 (12)	O2—C24—H24C	109.00
Fe1—C7—C8	69.83 (11)	H24A—C24—H24B	109.00
C6—C7—C8	108.16 (17)	H24A—C24—H24C	110.00
Fe1—C8—C7	69.50 (11)	H24B—C24—H24C	109.00
			
C2—Fe1—C1—C5	−119.32 (14)	C2—Fe1—C8—C9	149.66 (11)
C2—Fe1—C1—C11	120.12 (19)	C3—Fe1—C8—C7	−135.19 (12)
C3—Fe1—C1—C2	38.73 (10)	C3—Fe1—C8—C9	106.12 (12)
C3—Fe1—C1—C5	−80.58 (11)	C4—Fe1—C8—C7	−173.40 (11)
C3—Fe1—C1—C11	158.85 (17)	C4—Fe1—C8—C9	67.91 (15)
C4—Fe1—C1—C2	82.19 (11)	C6—Fe1—C8—C7	37.77 (12)
C4—Fe1—C1—C5	−37.13 (11)	C6—Fe1—C8—C9	−80.93 (13)
C4—Fe1—C1—C11	−157.69 (17)	C7—Fe1—C8—C9	−118.70 (17)
C5—Fe1—C1—C2	119.32 (14)	C9—Fe1—C8—C7	118.70 (17)
C5—Fe1—C1—C11	−120.57 (19)	C10—Fe1—C8—C7	81.30 (12)
C6—Fe1—C1—C2	−132.83 (10)	C10—Fe1—C8—C9	−37.40 (12)
C6—Fe1—C1—C5	107.86 (11)	C2—Fe1—C9—C8	−53.32 (18)
C6—Fe1—C1—C11	−12.71 (17)	C2—Fe1—C9—C10	−172.81 (12)
C7—Fe1—C1—C2	−88.80 (11)	C3—Fe1—C9—C8	−89.67 (13)
C7—Fe1—C1—C5	151.88 (11)	C3—Fe1—C9—C10	150.84 (11)
C7—Fe1—C1—C11	31.32 (17)	C4—Fe1—C9—C8	−132.66 (12)
C8—Fe1—C1—C2	−51.10 (17)	C4—Fe1—C9—C10	107.85 (12)
C8—Fe1—C1—C5	−170.41 (13)	C5—Fe1—C9—C8	−170.83 (12)
C8—Fe1—C1—C11	69.0 (2)	C5—Fe1—C9—C10	69.68 (15)
C10—Fe1—C1—C2	−172.79 (10)	C6—Fe1—C9—C8	81.92 (13)
C10—Fe1—C1—C5	67.89 (14)	C6—Fe1—C9—C10	−37.58 (12)
C10—Fe1—C1—C11	−52.67 (19)	C7—Fe1—C9—C8	38.04 (12)
C1—Fe1—C2—P1	−119.40 (15)	C7—Fe1—C9—C10	−81.46 (12)
C1—Fe1—C2—C3	117.41 (14)	C8—Fe1—C9—C10	−119.49 (17)
C3—Fe1—C2—P1	123.19 (16)	C10—Fe1—C9—C8	119.49 (17)
C3—Fe1—C2—C1	−117.41 (14)	C1—Fe1—C10—C6	68.19 (15)
C4—Fe1—C2—P1	159.87 (13)	C1—Fe1—C10—C9	−172.69 (11)
C4—Fe1—C2—C1	−80.73 (11)	C3—Fe1—C10—C6	−171.06 (13)
C4—Fe1—C2—C3	36.68 (11)	C3—Fe1—C10—C9	−51.93 (18)
C5—Fe1—C2—P1	−157.19 (13)	C4—Fe1—C10—C6	151.95 (12)
C5—Fe1—C2—C1	−37.79 (10)	C4—Fe1—C10—C9	−88.93 (13)
C5—Fe1—C2—C3	79.62 (11)	C5—Fe1—C10—C6	108.73 (12)
C6—Fe1—C2—P1	−48.55 (16)	C5—Fe1—C10—C9	−132.15 (12)
C6—Fe1—C2—C1	70.85 (13)	C6—Fe1—C10—C9	119.12 (17)
C6—Fe1—C2—C3	−171.74 (11)	C7—Fe1—C10—C6	−37.59 (12)
C7—Fe1—C2—P1	−10.52 (13)	C7—Fe1—C10—C9	81.53 (12)
C7—Fe1—C2—C1	108.88 (11)	C8—Fe1—C10—C6	−81.57 (13)
C7—Fe1—C2—C3	−133.71 (11)	C8—Fe1—C10—C9	37.55 (12)
C8—Fe1—C2—P1	32.86 (13)	C9—Fe1—C10—C6	−119.12 (17)
C8—Fe1—C2—C1	152.26 (11)	C12—P1—C2—Fe1	178.79 (10)
C8—Fe1—C2—C3	−90.33 (12)	C12—P1—C2—C1	93.31 (16)
C9—Fe1—C2—P1	67.43 (16)	C12—P1—C2—C3	−92.55 (17)
C9—Fe1—C2—C1	−173.17 (12)	C18—P1—C2—Fe1	−80.43 (12)
C9—Fe1—C2—C3	−55.76 (16)	C18—P1—C2—C1	−165.91 (15)
C1—Fe1—C3—C2	−39.27 (10)	C18—P1—C2—C3	8.23 (18)
C1—Fe1—C3—C4	81.23 (11)	C2—P1—C12—C13	173.55 (14)
C2—Fe1—C3—C4	120.50 (15)	C2—P1—C12—C17	−6.04 (18)
C4—Fe1—C3—C2	−120.50 (15)	C18—P1—C12—C13	70.72 (15)
C5—Fe1—C3—C2	−83.75 (11)	C18—P1—C12—C17	−108.86 (16)
C5—Fe1—C3—C4	36.75 (11)	C2—P1—C18—C19	113.07 (16)
C7—Fe1—C3—C2	67.35 (14)	C2—P1—C18—C23	−69.12 (17)
C7—Fe1—C3—C4	−172.15 (11)	C12—P1—C18—C19	−143.39 (16)
C8—Fe1—C3—C2	105.54 (12)	C12—P1—C18—C23	34.41 (17)
C8—Fe1—C3—C4	−133.96 (12)	C24—O2—C11—O1	−1.7 (3)
C9—Fe1—C3—C2	148.52 (11)	C24—O2—C11—C1	176.49 (16)
C9—Fe1—C3—C4	−90.98 (12)	Fe1—C1—C2—P1	114.97 (13)
C10—Fe1—C3—C2	−177.65 (13)	Fe1—C1—C2—C3	−60.24 (12)
C10—Fe1—C3—C4	−57.15 (17)	C5—C1—C2—Fe1	59.74 (12)
C1—Fe1—C4—C3	−82.20 (11)	C5—C1—C2—P1	174.71 (13)
C1—Fe1—C4—C5	38.18 (11)	C5—C1—C2—C3	−0.5 (2)
C2—Fe1—C4—C3	−37.33 (11)	C11—C1—C2—Fe1	−120.49 (18)
C2—Fe1—C4—C5	83.05 (11)	C11—C1—C2—P1	−5.5 (3)
C3—Fe1—C4—C5	120.38 (15)	C11—C1—C2—C3	179.27 (17)
C5—Fe1—C4—C3	−120.38 (15)	Fe1—C1—C5—C4	59.91 (13)
C6—Fe1—C4—C3	−177.45 (14)	C2—C1—C5—Fe1	−59.37 (12)
C6—Fe1—C4—C5	−57.07 (18)	C2—C1—C5—C4	0.5 (2)
C8—Fe1—C4—C3	65.71 (14)	C11—C1—C5—Fe1	120.86 (18)
C8—Fe1—C4—C5	−173.91 (11)	C11—C1—C5—C4	−179.23 (17)
C9—Fe1—C4—C3	105.12 (12)	Fe1—C1—C11—O1	−102.1 (2)
C9—Fe1—C4—C5	−134.50 (11)	Fe1—C1—C11—O2	79.71 (18)
C10—Fe1—C4—C3	148.17 (11)	C2—C1—C11—O1	−12.5 (3)
C10—Fe1—C4—C5	−91.45 (12)	C2—C1—C11—O2	169.40 (16)
C1—Fe1—C5—C4	−119.29 (15)	C5—C1—C11—O1	167.28 (19)
C2—Fe1—C5—C1	38.17 (10)	C5—C1—C11—O2	−10.9 (2)
C2—Fe1—C5—C4	−81.13 (11)	Fe1—C2—C3—C4	−59.22 (13)
C3—Fe1—C5—C1	82.31 (11)	P1—C2—C3—Fe1	−115.54 (14)
C3—Fe1—C5—C4	−36.99 (11)	P1—C2—C3—C4	−174.76 (14)
C4—Fe1—C5—C1	119.29 (15)	C1—C2—C3—Fe1	59.50 (12)
C6—Fe1—C5—C1	−91.24 (12)	C1—C2—C3—C4	0.3 (2)
C6—Fe1—C5—C4	149.47 (11)	Fe1—C3—C4—C5	−58.30 (13)
C7—Fe1—C5—C1	−54.20 (18)	C2—C3—C4—Fe1	58.35 (13)
C7—Fe1—C5—C4	−173.49 (14)	C2—C3—C4—C5	0.1 (2)
C9—Fe1—C5—C1	−175.02 (11)	Fe1—C4—C5—C1	−58.63 (13)
C9—Fe1—C5—C4	65.68 (14)	C3—C4—C5—Fe1	58.27 (13)
C10—Fe1—C5—C1	−134.72 (11)	C3—C4—C5—C1	−0.4 (2)
C10—Fe1—C5—C4	105.98 (12)	Fe1—C6—C7—C8	59.81 (14)
C1—Fe1—C6—C7	106.25 (12)	C10—C6—C7—Fe1	−59.48 (14)
C1—Fe1—C6—C10	−134.30 (11)	C10—C6—C7—C8	0.3 (2)
C2—Fe1—C6—C7	64.04 (15)	Fe1—C6—C10—C9	−59.21 (14)
C2—Fe1—C6—C10	−176.51 (11)	C7—C6—C10—Fe1	58.91 (14)
C4—Fe1—C6—C7	−171.54 (13)	C7—C6—C10—C9	−0.3 (2)
C4—Fe1—C6—C10	−52.09 (19)	Fe1—C7—C8—C9	59.88 (14)
C5—Fe1—C6—C7	151.09 (11)	C6—C7—C8—Fe1	−60.11 (14)
C5—Fe1—C6—C10	−89.47 (13)	C6—C7—C8—C9	−0.2 (2)
C7—Fe1—C6—C10	119.45 (17)	Fe1—C8—C9—C10	59.59 (14)
C8—Fe1—C6—C7	−37.90 (12)	C7—C8—C9—Fe1	−59.56 (13)
C8—Fe1—C6—C10	81.55 (13)	C7—C8—C9—C10	0.0 (2)
C9—Fe1—C6—C7	−81.78 (12)	Fe1—C9—C10—C6	59.32 (14)
C9—Fe1—C6—C10	37.67 (12)	C8—C9—C10—Fe1	−59.15 (14)
C10—Fe1—C6—C7	−119.45 (17)	C8—C9—C10—C6	0.2 (2)
C1—Fe1—C7—C6	−92.09 (12)	P1—C12—C13—C14	−177.91 (14)
C1—Fe1—C7—C8	148.96 (11)	C17—C12—C13—C14	1.7 (3)
C2—Fe1—C7—C6	−136.55 (11)	P1—C12—C17—C16	178.41 (15)
C2—Fe1—C7—C8	104.49 (12)	C13—C12—C17—C16	−1.2 (3)
C3—Fe1—C7—C6	−176.28 (11)	C12—C13—C14—C15	−0.9 (3)
C3—Fe1—C7—C8	64.77 (15)	C13—C14—C15—C16	−0.6 (3)
C5—Fe1—C7—C6	−55.55 (19)	C14—C15—C16—C17	1.1 (3)
C5—Fe1—C7—C8	−174.50 (14)	C15—C16—C17—C12	−0.2 (3)
C6—Fe1—C7—C8	−118.95 (16)	P1—C18—C19—C20	178.32 (16)
C8—Fe1—C7—C6	118.95 (16)	C23—C18—C19—C20	0.4 (3)
C9—Fe1—C7—C6	81.04 (13)	P1—C18—C23—C22	−177.83 (16)
C9—Fe1—C7—C8	−37.92 (12)	C19—C18—C23—C22	0.0 (3)
C10—Fe1—C7—C6	37.36 (11)	C18—C19—C20—C21	0.0 (3)
C10—Fe1—C7—C8	−81.59 (12)	C19—C20—C21—C22	−0.9 (3)
C1—Fe1—C8—C7	−57.39 (18)	C20—C21—C22—C23	1.3 (3)
C1—Fe1—C8—C9	−176.09 (13)	C21—C22—C23—C18	−0.8 (3)
C2—Fe1—C8—C7	−91.65 (12)		

### Hydrogen-bond geometry (Å, °)

**Table:**

*D*—H···*A*	*D*—H	H···*A*	*D*···*A*	*D*—H···*A*
C4—H4···O1^i^	0.93	2.58	3.184 (2)	123
C15—H15···O1^ii^	0.93	2.58	3.494 (3)	167

Symmetry codes: (i) −*x*+1/2, −*y*, *z*+1/2; (ii) *x*−1/2, −*y*−1/2, −*z*+1.
